# P34 HCV genotype diversity in patients with pulmonary TB

**DOI:** 10.1093/jacamr/dlaf230.041

**Published:** 2025-12-04

**Authors:** Irina Lizinfeld, Liubov Parolina, Natalia Pshenichnaya, Larisa Davidova, Vadim Testov, Irina Vasilyeva

**Affiliations:** Federal State Budgetary Institution «National Medical Research Center of Phthisiopulmonology and Infectious Diseases» of the Ministry of Health of the Russian Federation (NMRC PhPI), Moscow, Russia; Federal State Budgetary Institution «National Medical Research Center of Phthisiopulmonology and Infectious Diseases» of the Ministry of Health of the Russian Federation (NMRC PhPI), Moscow, Russia; Federal Budget Institute of Science «Central Research Institute of Epidemiology», Moscow, Russian Federation; Federal State Budgetary Institution «National Medical Research Center of Phthisiopulmonology and Infectious Diseases» of the Ministry of Health of the Russian Federation (NMRC PhPI), Moscow, Russia; Federal State Budgetary Institution «National Medical Research Center of Phthisiopulmonology and Infectious Diseases» of the Ministry of Health of the Russian Federation (NMRC PhPI), Moscow, Russia; Federal State Budgetary Institution «National Medical Research Center of Phthisiopulmonology and Infectious Diseases» of the Ministry of Health of the Russian Federation (NMRC PhPI), Moscow, Russia

## Abstract

**Background:**

Chronic HCV and pulmonary TB are widespread infectious diseases with a high socio-economic burden. Determining the HCV genotype in TB patients is of key importance, since comorbid pathology poses challenges for combined therapy and affects disease prognosis, while different genotypes respond differently to direct-acting antivirals (DAAs).

**Objective:**

. To identify the types of HCV genotypes in patients with pulmonary TB and to characterize the clinical, demographic and social profile of patients with combined pathology.

**Materials and methods:**

. A retrospective, cross-sectional, descriptive study was performed using data from the Federal Hepatitis Registry. Seventy patients who met the inclusion criteria were included: confirmed coinfection with HCV and pulmonary TB, available HCV genotyping results and an established diagnosis of TB. Descriptive statistics were used to summarize the data. Categorical variables are presented as frequencies and percentages, while continuous variables are reported as mean ± SD.

**Results:**

The most frequently detected HCV genotype was 3a (49 patients, 70.0%), followed by genotype 1b (12 patients, 17.1%), genotype 1a (8 patients, 11.4%) and genotype 2 (1 patient, 1.4%). Men accounted for 55 patients (78.6%) and women, 15 (21.4%). The mean age was 44.1 ± 9.0 years, with the largest proportion aged 30–44 years (30, 42.9%) and 45–59 years (33, 47.1%) and the smallest proportion aged 0–17 years (2, 2.9%), 18–29 years (1, 1.4%) and over 60 years (4, 5.7%). According to TB forms based on WHO/CDC classification, the distribution was as follows: pulmonary TB, bacteriologically confirmed: 37 patients (52.9%); pulmonary TB, clinically diagnosed: 21 patients (30.0%); extrapulmonary TB: 9 patients (12.8%); miliary TB: 3 patients (4.3%). Analysis of the patients’ social status revealed that 44 (62.8%) were unemployed, 17 (24.3%) were employed, 1 (1.4%) was a serviceman, 2 (2.9%) were students or schoolchildren, 2 (2.9%) were incarcerated individuals and 4 (5.7%) were retirees. Disability was noted in 10 patients (14.3%) and a history of antiviral therapy was reported in 6 patients (8.6%). In patients with HCV coinfection and pulmonary TB, genotype 3a predominates among working-age men. Genotype 3a is associated with faster liver disease progression, development of fibrosis, cirrhosis and metabolic disorders, highlighting the need for early detection and careful monitoring. Bacteriologically confirmed TB was most common, while some patients had unconfirmed diagnoses. A significant proportion of patients belonged to socially vulnerable groups, particularly the unemployed, emphasizing the need for social support and educational programmes to improve therapy adherence and access to medical care. The data underscore the importance of HCV genotyping at the time of coinfection diagnosis before starting antiviral therapy, conducting targeted HCV screening among patients with TB and ensuring access to direct-acting antiviral treatment tailored to genotype and treatment duration, especially for patients with cirrhosis.

**Conclusions:**

In patients with coinfection of HCV and pulmonary TB , genotype 3a predominated, with men of working age and socially vulnerable groups more frequently affected, highlighting the need for early genotyping, comprehensive management and access to antiviral therapy considering genotype and social status.

